# Cellular, Antibody and Cytokine Pathways in Children with Acute SARS-CoV-2 Infection and MIS-C—Can We Match the Puzzle?

**DOI:** 10.3390/antib11020025

**Published:** 2022-04-01

**Authors:** Snezhina Lazova, Yulia Dimitrova, Diana Hristova, Iren Tzotcheva, Tsvetelina Velikova

**Affiliations:** 1 Pediatric Department, University Hospital “N. I. Pirogov”, 21 “General Eduard I. Totleben”, Blvd., 1463 Sofia, Bulgaria; julia.dimitrova@pirogov.bg (Y.D.); iren.tzotcheva@pirogov.bg (I.T.); 2 Health Care Department, Faculty of Public Health, Medical University Sofia, Bialo More, 8 Str., 1527 Sofia, Bulgaria; 3 Department of Immunology, National Center of Infectious and Parasitic Diseases, 1504 Sofia, Bulgaria; did_hris@abv.bg; 4 Department of Clinical Immunology, University Hospital Lozenetz, Sofia University St. Kliment Ohridski, Kozyak 1 Str., 1407 Sofia, Bulgaria; tsvelikova@medfac.mu-sofia.bg

**Keywords:** SARS-CoV-2, MIS-C, COVID-19, cytokine, cell-mediated immunity, antibodies, SARS-CoV-2 specific immunity, T cells, B cells

## Abstract

The newly identified strain of the Coronaviridae family called severe acute respiratory syndrome (SARS-CoV-2) recently became the most significant health threat for adults and children. Some main predictors of severe clinical course in patients with SARS-CoV-2 infection are age and concomitant health conditions. Therefore, the proper evaluation of SARS-CoV-2-specific immunity is urgently required to understand and predict the spectrum of possible clinical phenotypes and recommend vaccination options and regimens in children. Furthermore, it is critical to characterize the nature of SARS-CoV-2-specific immune responses in children following asymptomatic infection and COVID-19 and other related conditions such as multisystem inflammatory syndrome (MIS-C), para-infectious and late postinfectious consequences. Recent studies involving children revealed a variety of cytokines, T cells and antibody responses in the pathogenesis of the disease. Moreover, different clinical scenarios in children were observed-asymptomatic seroprevalence, acute SARS-CoV-2 infection, and rarely severe COVID-19 with typical cytokine storm, MIS-C, long COVID-19, etc. Therefore, to gain a better clinical view, adequate diagnostic criteria and treatment algorithms, it is essential to create a realistic picture of the immunological puzzle of SARS-CoV-2 infection in different age groups. Finally, it was demonstrated that children may exert a potent and prolonged adaptive anti-SARS-CoV-2 immune response, with significant cross-reactions against other human Corona Viruses, that might contribute to disease sparing effect in this age range. However, the immunopathology of the virus has to be elucidated first.

## 1. Introduction

The newly identified strain of the Coronaviridae family called severe acute respiratory syndrome (SARS-CoV-2; formerly called 2019-nCoV) has the ability to infect the human body and to cause a recently described infectious disease called coronavirus disease 19 (COVID-19) [1]. Coronaviride is a family of (+) RNA viruses with an envelope associated with primarily respiratory and fecal-oral transmitted infections. This virus family is characterized by the significant genome content, infecting amphibians, birds, and mammals [1].

Soon after the first SARS-CoV-2 identification during the uncommon respiratory outbreak in China and the first WHO report, COVID-19 was considered a global pandemic on 11 March 2020 [1,2]. The following two years confirmed the expectations that we are witnessing one of the deadliest pandemics in history [1].

It is known that SARS-CoV-2 enters the human target cells after interaction of the virus S protein with an angiotensin-converting enzyme (ACE) 2 host cell receptors and processing of S protein with endogenous transmembrane serine protease 2 (TMPRSS2) [3]. This interaction is followed by endocytosis, viral RNA release, replication and translation into viral proteins and new viral particles release [3].

Although it was thought that the disease is milder in the youngest population, COVID-19 affects both adults and children [4]. Indeed, the vast accumulated data confirmed that the incidence and prevalence of COVID-19 in children resemble those in adults [4].

Another recently described nosology associated with SARS-CoV-2 infection is the multisystem inflammatory syndrome in children (MIS-C) [4]. Quite similar to the vasculitis Kawasaki disease (KD), MIS-C was recognized as a separate disease a few months after the pandemic onset. However, rapidly accumulating data on MIS cases in children from around the world convincingly link the disease to the new coronavirus [4,5,6,7]. This systemic inflammation may involve multiple organs and systems, particularly the heart, gastrointestinal system, skin, eyes, kidneys, lungs and brain. One of the prominent features of MIS-C is the delay of symptoms onset, usually at least 14 days after SARS-CoV-2 infection [4].

Three leading healthcare organizations, the World Health Organization (WHO) [5], the Centers for Disease Control and Prevention (CDC, Atlanta, GA, USA) [6] and the Royal College of pediatrics and child health (RCPCH, London, UK) [7], had published recommendations and criteria for MIS-C diagnosis. Therefore, strict adoption and following the internationally accepted diagnostic criteria are recommended. In such a way, we can collect qualitative and quantitative relevant knowledge to highlight the main immunological mechanisms that unlock or trigger the condition in certain patients. The diagnostic criteria of MIS-C [5,6,7] are summarized in Table 1.

The three reliable and official sources presented in Table 1 include a few immunological entries amongst the MIS-C criteria. The first published case reports of unusual childhood multisystem inflammatory conditions possibly associated with SARS-CoV-2 infection date from April 2020 [8]. WHO includes immune-related fever more than 3 days, mucosal and cutaneous inflammation signs (oral, hands, or feet) and elevated markers of systemic inflammation (e.g., ESR, CRP, or procalcitonin) [5]. Amongst the criteria of RCPCH (UK) are fever > 38.5 °C lasting more than 24 h, lymphadenopathy, mucus membrane changes, swelling, lymphopenia, neutrophilia in most of the children, high CRP, IL-10 (if available), IL-6 (if available) [7]. CDC (US) also includes some immunological entries among the criteria—fever and laboratory evidence of inflammation [7].

Some children manifest an overlapping with the KD to varying degrees according to these criteria [9]. However, any case that meets the definition and fulfills the criteria, as well as any childhood dead with proven SARS-CoV-2 infection, should be considered and reported as MIS-C [4]. It is accepted that positive serology for SARS-CoV-2 is enough informative diagnostic criteria for MIS-C, assuring the previous infection with the virus followed by immune reactions, keeping in mind the widespread of COVID-19 in the community. However, that statement should probably be reconsidered soon, especially in the context of the new virus mutants of concern, variants that affect children on a larger scale and the extended indications for childhood vaccination. Moreover, solving the immunological puzzle in MIS-C can shortly provide new, more reliable diagnostic criteria.

Despite the tremendous progress and the conduct of in-depth research, the pathophysiological mechanism of MIS-C still remains unclear [10]. The general immunity fitness cannot explain the different disease manifestations. However, recent data showed increased IL-6 production in the more severe cases [10]. This suggests that MIS-C is a matter of immune pathological dysregulation [10].

A characteristic laboratory constellation in MIS-C patients includes reduced lymphocyte and reduced or normal thrombocyte count [11]. Typical for the acute phase of MIS-C is the increased inflammation state. The latter is objected by detecting high levels of proinflammatory cytokines, such as IL-6, IL-8, tumor necrosis factor-α (TNF-α), interleukin-1β (IL-1β), IL-10, IL-17, interferon-γ (IFN-γ), IL-2 receptor agonist and other molecules and acute-phase proteins like C-reactive protein (CRP), procalcitonin and ferritin [11]. In addition, elevations of the N-terminal pro-B-type natriuretic peptide (NT-proBNP) and troponin, which are markers of myocardial dysfunction and damage, have been reported [10,11]. All these outcomes confirm the immunological mechanisms in MIS-C as a consequence of usually mild or asymptomatic previous COVID-19 in children.

Similar to KD, which goes with no proven etiological cause, the link between MIS-C and the new coronavirus has not been conclusively confirmed [12]. However, there is an obvious epidemiological causal link between the emergence of the new highly contagious pathogen SARS-CoV-2 and emerging cases of a relatively unknown condition with its own clinical and morphological characteristics, although similar to preexisting and described diseases [12]. Moreover, with the pandemic development, the cases of MIS-C followed local epidemics with acute SARS-CoV-2. Meanwhile, the scientific community has the issue of analyzing the effects of new strains and variants of concern, the efficacy of anti-epidemic measures and the accumulation of a population that has repeatedly encountered the virus.

As we mentioned above, physicians have found some clinical similarities between MIS-C and KD [9]. KD is a disease of early childhood characterized by fever and inflammation of blood vessels that can lead to coronary artery aneurysms. In contrast to KD, MIS-C is represented with a procoagulant state due to increased fibrinogen and D-dimer levels and decreased platelet count [13].

Similar to COVID-19, lymphopenia affecting CD4+ (helper T) cells, CD8+ (cytotoxic T) cells and γδ T cells was observed at the beginning of the acute phase of MIS-C [13,14]. However, the number of total neutrophils, monocytes, dendritic cells and natural killer cells remains normal, despite changes in the expression of functional molecules. Reported elevated IL-8 levels along with IL-6 may be associated with increased neutrophil activation, which affects T and B lymphocyte responses [15]. To get an insight into the intimate pathogenetic and immunological mechanisms that push a child′s body in a multisystemic inflammatory state, it is essential to define possible immune response scenarios during spontaneous SARS-CoV-2 infection. In turn, this would allow the discovery of new reliable diagnostic and prognostic factors to identify risk groups and promptly define all possible clinical phenotypes of MIS-C. Furthermore, having systematized in-depth knowledge on the MIS-C could guide the prevention strategies, including vaccination.

## 2. Immunology of MIS-C

When a new disease such as COVID-19 emerges, the strategies initially focus on monitoring and testing patients with severe illness. Additionally, molecular tests are used to measure acute infections, as health care seek and need this. In contrast, data on molecular pathogenesis and interactions of the virus with the immune system remain insufficient [15,16]. In this way, we can often miss some mild or asymptomatic infections that do not require medical attention. Still, from an epidemiological point of view, children are also involved in infection transmission [17].

As we already mentioned, in youngsters, we often observe asymptomatic or mild to moderate SARS-CoV-2 infection, in contrast to high hospitalization and mortality rates in older adults [18]. As a result, there is a great deal of scientific curiosity and attention in determining immune reactions, pathways and mechanisms to SARS-CoV-2 in children [19]. So far, such investigations have been limited. Still, compared to adult patients, they indicate a deficiency of the nucleocapsid-specific antibody responses and diminished antibody and cellular responses during or shortly after infection [20,21].

### 2.1. Innate Immune Response to SARS-CoV-2

As we mentioned above, the SARS-CoV-2 entrance and invasion usually begin at the level of human nasopharyngeal cells. There, the viral S protein interacts successively with the host ACE2 receptor and transmembrane protease serine 2 (TMPRSS2) [8]. In addition, however, the host cells and innate immune cells employ a variety of receptors associated with the first line of defense [22]. Such receptors are pattern recognition receptors (PRRs) expressed not only on the surface of numerous hosts′ innate immune cells but also in phagocytic vesicles and the cytoplasm. These receptors serve for the early immune recognition of different pathogen-associated molecular patterns (PAMPs) [22].

The most well-studied PRRs include toll-like receptors (TLRs), retinoic acid-inducible gene 1 (RIG-I)–like receptors (RLRs) and nucleotide oligomerization domain (NOD)–like receptors (NLRs). TLRs are the most effective PRRs. Their family includes ten representatives in a human cell—TLR1 to TLR10. TLRs, RLRs, and NLRs are related mostly to viral recognition [23,24]. Other known PRRs are C-type lectin receptors (CLRs), scavenger receptors, N-formyl methionyl-leucyl-phenylalanine [23].

In the case of SARS-CoV-2 contamination, the first defense line of the innate immunity is the surface membrane located PRR. If they fail, cytoplasm PRRs like MDA-5 and RIG-I can detect the viral agent [23,24,25]. Table 2 summarizes the currently known receptors involved in the anti-SARS-CoV innate immune defense. At this point of knowledge gathered, there is no difference in these innate mechanisms in children and adults during COVID-19.

A vast number of innate receptors are found to be involved in SARS-CoV-2 infection. It is known that TLRs can identify viruses like SARS-CoV and MERS-CoV and possibly their identical SARS-CoV-2. Experimental coronavirus infection models described increased TLR3 expression and consequent generation of IFN type I as well as proinflammatory cytokines [23,24]. On the other hand, it is well known that activating Myeloid differentiation primary response 88 (MYD88) and TIR-domain-containing adapter-inducing interferon-β (TRIF) lead to inhibiting adapter proteins consecutively after the TLRs linking. TRIF is related to type-I interferons production, which can modify immunological responses [23]. Furthermore, the MYD88 pathway induces the production of several groups of proinflammatory cytokines and is considered a primary response to SARS-CoV-2 [23,24].

The RLRs family members serve as cytosol defense that reacts to a viral invasion by enhancing the production of type I interferons. Three members belong to this receptors family: RIG-I, laboratory of genetics and physiology 2 (LGP2) and melanoma differentiation-associated gene 5 (MDA5) [23]. RLRs are widely present in the cytosol; therefore, they can recognize endosomal escaping viruses infecting by direct cell membrane fusion. It is documented that MDA5-deficient mice are incredibly vulnerable to ssRNA viral infections such as SARS-CoV, MERS-CoV, SARS-CoV-2, rhinoviruses, and coxsackieviruses B [23].

DC-SIGN (dendritic cell-specific intercellular adhesion molecule-3 grabbing non-integrin) belongs to the C-type lectin innate immune response receptors group [26]. Very high expression of these receptors is detected on the surface of immature DCs. Another receptor called L-SIGN (liver/lymph node-specific SIGN) is a DC-SIGN homolog [26]. This protein is expressed in many tissues such as the liver, lymph node, placenta and may induce subsequent SARS-CoV-2 invasion of these tissue cells. In addition, monocytes, respiratory macrophages, endothelial cells, and preliminary DCs can express C-type lectin markers [23].

DC-SIGN was proposed by Zhang et al. as a marker for viral spread from one organ to another [26]. Although these receptors can serve as an alternative to ACE-2 by connecting to the RBD S-protein domain, their affinity is lower [26]. Nevertheless, in some studies, DC-SIGN is described as an ACE-2 receptor cofactor because of its function in viral absorption and transmission to ACE-2-expressing target cells [23].

Han et al. discovered that SARS-CoV-2 glycans on the S protein affect the type of receptor-mediated infection [27]. Different S protein glucans are essential for viral penetration via the DC-SIGN and ACE-2. DC-SIGN and L-SIGN have a synergistic role and act as significant receptors for SARS-CoV-2 depending on the S protein glycosylation. In addition, they are involved in viral phagocytosis [23,24,25].

**Figure 1:**
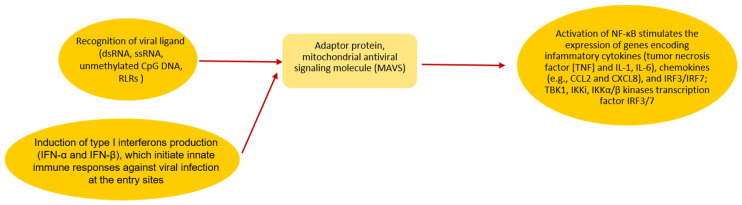
During SARS-CoV-2 invasion in children and adults, the virus and the host cell fuse their membranes mediated by an interaction between the coronaviral S protein and ACE2 receptor on the host cells followed by releasing its RNA into the cytosol [28]. Activation of RIG-I or MDA-5 starts signaling through an adaptor protein, mitochondrial antiviral signaling molecule (MAVS) [28]. It subsequently stimulates the activation of TBK1, Ikki, IKKα/β kinases and transcription factor IRF3/7 and NF-κB. The next step is IFNs expression and interferon-stimulated genes (ISGs) expression and the expression of inflammatory cytokines [23].

Schematic SARS-CoV-2 invasion in both children and adults is presented in Figure 1. Some of the mechanisms involving TLRs [28] are presented.

Additionally, Yu-Zhi et al. report that SARS-CoV-2 disrupts mitochondrial antiviral signaling molecule (MAVS) aggregation [29].

Interferons are released from infected cells to prevent viral spread from cell to cell to provide immunomodulatory effects [23]. Mechanisms by which viruses can block the IFN response were assessed in vitro. First, in the initial stage of viral infection, the immune system generates IFN-I [30]. Then, the early IFN response to SARS-CoV-2 induces antiviral activity and suppresses the subsequent development of the disease. Otherwise, delayed IFN-I responses are associated with the overactivation of proinflammatory responses.

Additionally, coronaviruses can disrupt PRR stimulation and IFN signaling [22,31]. For example, patients with severe COVID-19 have decreased IFN type I and III secretion. This may result from a reduction in the initial IFN response, leading to uncontrolled and prolonged inflammation in a time-dependent manner [22,23]. Early disrupted IFN-I response determines the SARS-CoV-2 infection [32,33]. Interestingly, the expression of IFN-stimulated genes (ISGs) is significantly higher in patients with COVID-19 compared to MIS-C [30].

Here we have to highlight that children possess a pre-activated innate immune response associated with early production of IFNs in infected airways [23]. As a result, the clearance of the virus is faster than in adults. This can lead to lower viral replication and load. Loske et al. associated this observation with the high expression of genes encoding RIG-I, MDA5 and LGP2 in children [25].

**Figure 2:**
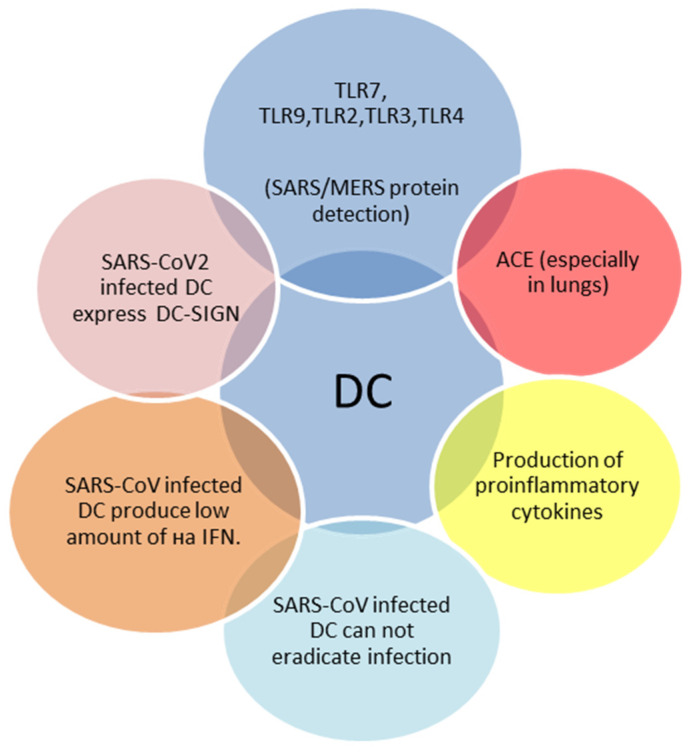
Main features of SARS-CoV-2 infected dendritic cells (DCs) [23,32,33,34].

Dendritic cells (DCs) are professional antigen-presenting cells that serve as a bridge between innate and adaptive immune responses. They also produce interferon type I (IFN) in response to TLR7 and TLR9 activation [32]. Two subtypes of DCs are known: “conventional” (cDCs) and plasmacytoid (pDCs) [33]. In adults, pDCs produce large amounts of interferon type I (IFN) upon PRRs activation. In contrast, neonatal pDCs are severely limited in interferon secretion in response to various viruses [34]. This may explain age-dependent features of pathological pathways of viral infections. The role of DCs in SARS-CoV-2 infection in both adults and children is presented in Figure 2.

Monocytes and macrophages are also part of the first defense line against pathogens. Therefore, they are essential for infection control [35]. The primary monocytes and macrophages′ roles during SARS-CoV-2-provoked immune response are presented in Table 3.

During SARS-CoV-2 infection, monocytes and macrophages accumulate into the lungs [23]. Macrophage activation syndrome (MAS) development, one of the characteristics of a cytokine storm, depends on inflammatory cytokines such as TNF-α and IL-6 [38]. Such dysregulation may be due to the involvement of different subsets of monocytes in successive stages of the disease. For example, in patients with COVID-19, the number of CD14++CD16− T lymphocytes is typically low, while CD14++CD16+ (intermediate) and CD14+CD16++ (non-classical) monocytes increase [38,39]. Recently described CD56+CD14+Ki67+IFN-γ+ monocyte may play a crucial role in severe COVID-19 [40].

To sum up, immunological characteristics of patients with MIS-C are similar to patients with severe COVID-19. However, the expression of IL-6, IL-10, IL-18, TNF-a, MCP1, IL-1RA and sCD25 may be even higher in MIS-C [41]. As a rule, children have a milder course of COVID-19, probably due to lower expression of ACE2 receptors [42,43]. Additionally, Park and Iwasaki indicated that PRRs and the IFN type I and III are critical players for successfully resolving SARS-CoV-2 infection [44], and levels of IFN-α2, IFN-γ, IP-10, IL-8 and IL-1β are higher in nasal secretions in children [43]. Furthermore, Loske et al. found IFN dependent basal expression of innate receptors (MDA5 (IFIH1), RIG-I (DDX58), etc.) in epithelial cells of the upper respiratory tract, higher in children than in adults [25]. Furthermore, each individual′s unique innate receptors signature may be associated with severe COVID-19. A possible explanation for this abnormal expression pattern is the impaired IFN signaling pathway in adults but not children [45,46,47].

### 2.2. Adaptive Immune Responses to SARS-CoV-2

In young children, there are more naïve T-cells ready to recognize new infectious agents, such as SARS-CoV-2, than in adults. Simultaneously, children′s T memory cells are primed to react to other common coronaviruses because they have recently encountered such pathogens [14]. A subgroup of cytotoxic T cells (CTL2), called KLRC1 (NKG2A)+, was experimentally demonstrated by Loske et al. [25]. Moreover, this lectin-like receptor (NKG2A) located on the cytotoxic T cells has inhibitory functions during childhood and reduces the chances of immune hyperactivation. Meanwhile, it prevents apoptosis and sustains the virus-specific CD8+ T cells, which express a high level of cytotoxic mediators in the absence of viral infection [12]. In adults, during SARS-CoV-2 infection, these cells secrete a lot of IFNγ. Upon infection, children secrete significantly higher levels of IFNγ than adults during the whole course of illness [12].

The cytotoxic capacity and the availability of such T cell subtypes can explain the better immune reaction against viruses in children. In addition, Cohen et al. identified a distinct CD8+ T cell memory type population (CD8 Tm) in children with SARS-CoV-2 infection that is almost absent in adults [21]. On the other hand, in preterm and newborn babies, monocytes and macrophages are immature with less TLR4 on their surfaces compared to adults. Consequently, these newborns have impaired phagocytosis and secretion of bioactive molecules [35]. The most extensive retrospective pediatric clinical study of COVID-19 from Dong et al. stated that the most severe rate of the disease is among children under 1 year. Indeed, low IgG and IgA levels were also observed among patients younger than 1 year old. Furthermore, IgG remains low until 2 years of age. Only elevated CD19+ B cell count levels were found among patients less than 6 years [18].

In contrast, A typical hallmark of aging is the involution of the thymus, with a natural dropout of production of naïve T cells and limited response to new pathogens. In addition, the T cell function is often disrupted. In adults, phagocytosis, respiratory burst and bacterial killing could be compromised, and the cytotoxicity of the NK cells [30]. Additionally, the immune triggers cannot activate the DCs at the required level, and the T cells′ production of IL-2 and IFNγ by the T cells is decreased. With aging, the production of antibodies from the B cells is altered, and the cytotoxic T-cell activity declines. All of these immune alterations are typical for the elderly. This can explain the considerable sensitivity to respiratory viruses like RSV, influenza, and SARS-CoV-2 [30].

In children, novel insights showed that the typical polyclonal Vβ21.3+ T cell expansions might be associated with MIS-C [40]. The activated phenotype of Vβ21.3+ T cells express high levels of CX3CR1, a marker of patrolling monocytes and cytotoxic lymphocytes. Moreover, the CX3CR1-CX3CL1 axis probably leads to vascular inflammation in MIS-C. Furthermore, it is possible that Vβ21.3+ CD4 and CD8 T cell expansions may be the distinctive marker of MIS-C compared to KD and COVID-19 [40].

CX3CR1 binding to CX3CL1 induces inflammation on the endothelial. Thus, one could assume that deep immune profiling of MIS-C shows marked although transient immune activation compared to COVID-19 in adults and children [48]. Moreover, Vβ21.3+ T cell expansions may be involved in the raised levels of some serum cytokines such as IL-18 and IL-1RA, a hallmark of the so-called cytokine storm.

Altered adapted immunity in MIS-C was also demonstrated by Ramaswamy [49]. They showed that some genes responsible for the cytotoxicity in NK and CD8+ T cells are overexpressed in children with MIS-C [49]. Moreover, the observed disorders of the immune system towards dysregulation and autoreactivity correlated with the severity of MIS-C.

All of these raise the assumption that Vβ21.3+ cell expansion may be driven by a superantigen structure in MIS-C. Superantigens can bind TCR in external regions and MHC molecules [50] and, thus, cause massive activation and proliferation of TCR Vβ chain-specific T cells. In contrast, classical antigens activate the proliferation of T cells with various Vβ. Since MIS-C is a condition developed following COVID-19, it is assumed that Vβ21.3+ T cell expansion is delayed compared to acute SARS-CoV-2 infection, even after the virus is not presented in the organism, but the inflammation is on the rise. In addition, Vβ-restricted T cells contribute to endothelial inflammation and vascular injury by adhering to endothelial cells [51].

Immune dysregulation in MIS-C associated with adaptive immunity also involves secondary autoimmune reactions and postinfection immune alteration. Several studies documented the presence of autoantibodies in the serum of MIS-C patients. Amongst them, some are against endothelial antigens [49]. Additionally, it was shown that these autoantibodies can form complexes with endogenous antigens that act as superantigens [50,51].

The postinfection immune dysregulation included the previously mentioned activation of CX3CR1þ CD8þ T cells in the vascular system, which are thought to play an essential role in endothelial inflammation and damage observed in MIS-C [48].

Magdalena Okarska-Napierała et al. phenotyped 31 MIS-C patients at three-time points: acute, convalescent, and recovery stages of the disease. They found a considerable alteration in lymphocyte numbers during MIS-C [52]. Furthermore, changes mainly affected T cells subsets and correlated strongly with the disease severity (such as hypotension, etc.). Some MIS-C patients develop transient lymphocytosis during the convalescence period [52].

Usually, in youngsters (median age 9 years), MIS-C appears 2–4 weeks after infection [53]. However, although the immunological foundation for this illness is unknown, it is distinguished by diffuse endothelium damage and widespread autoantibody production [54,55]. B-cells probably also contribute to the autoimmune reactions and pathogenesis of MIS-C by producing different autoantibodies, such as anti-Jo-1 and anti-La [56]. Also, there is a cross-reactivity between the virus and self-antigens. This usually results from autoantigen spread because of the massive tissue damage.

It is worth emphasizing that serological evaluation of anti-SARS-CoV-2 antibodies helps determine the size of the outbreak or the degree of infection spread in a population. Thus, seroprevalence studies provide a more comprehensive picture of what proportion of the population is infected with SARS-CoV-2 and capture unrecognized cases that have not been identified through routine or active surveillance [18].

Many ongoing studies aim to better understand the antibody response following SARS-CoV-2 infection. Studies showed that people infected with SARS-CoV-2 develop specific to the virus antibodies. However, levels of these antibodies can vary between those with milder disease or asymptomatic infection (lower levels of antibodies) and with severe disease (higher levels of antibodies). Therefore, most studies focused on the extent to which antibody levels are defensive and how long these antibodies last [18].

Similar to adults, antibody levels against coronaviruses decrease gradually over time in children. Despite declining titers, resistance due to neutralizing antibodies has been established for at least one year in recovered from MERS-CoV and SARS-CoV-1 patients [57]. However, there are no data for children from the last epidemics with coronaviruses. In adults, long-term persistence of T-cell responses has been reported for both SARS-CoV-1 and MERS-CoV, suggesting that decreasing antibody titers may not mean loss of immunity. Therefore, the relationship between the measured antibodies levels and the durability of the protection still remains elusive [57,58].

However, neutralizing antibodies may ensure better immune protection. Still, debate continues over whether the primary immune defense mechanism against SARS-CoV-2 infection is exerted by the neutralizing antibodies [59]. Cell-mediated immune mechanisms may be crucial in controlling SARS-CoV-1 and MERS-CoV viral load. Further studies are needed to elucidate the association between neutralizing antibodies, antibody-dependent cell-mediated immune responses and the desired seroprotection [57]. Regarding children, the data is far more insufficient.

The timing of exposure to the four other endemic human coronaviruses may be a driver of variable immune responses to SARS-CoV-2 across the life span (hCoVs) [60]. These include the beta-coronaviruses (OC43 and HKU-1), which have 38% and 35% homology with SARS-CoV-2, respectively, and the more distantly related alpha-coronaviruses (NL63 and 229E), which have about 31% amino acid similarity [61,62]. These coronaviruses produce common mild pediatric illnesses, with antibody seroconversion usually before five years old.

Alpha- or Beta-coronavirus infections lead to short-term immunity against coronavirus reinfection. In addition, it indicates temporary cross-reactive immunity between the coronaviridae family [60]. As a result, recent infection with hCoV may explain the presence of cross-reactive neutralizing antibodies in seronegative children. Immune responses to hCoV are probably maintained throughout life, although they do not give sterilizing immunity [61,62]. As a result, repeated infections are expected, raising concerns that a similar pattern would emerge following SARS-CoV-2 infection.

Antibody responses to spike protein after infection were enhanced in children. It was shown that seroconversion increased responses to seasonal Beta-coronaviruses via S2 domain cross-recognition [63]. The ability of children and adults to neutralize viral variants was comparable. Additionally, spike-specific T cell responses were enhanced two times in children, including in many seronegative youngsters, indicating preexisting cross-reactions to seasonal coronaviruses. Importantly, children sustained antibody and cellular responses at least 6 months following infection. Still, adults experienced relative fading of the immune responses [64]. Still, these differences in children′s and adults′ antibodies responses to coronaviruses do not explain the existence of MIS-C.

One important discovery was that the extent of the adaptive immunity to SARS-CoV-2 is more noteworthy in children than in adults. This contrasts with prior studies that found weaker T cell responses in children [21].

Dowell et al. also discovered that many children had cross-reactive specific T cells to coronaviruses before and after SARS-CoV-2 infection [64]. Similarly, SARS-CoV-2-specific T cells were observed in more than half of seronegative children, including samples collected before infection. Therefore, it is assumed that these coronaviruses-specific T cells are cross-reactive to SARS-CoV-2 peptides [65,66].

It has also been shown that in the early postinfection period, children do not produce efficient antibodies against nucleocapsid (N antigen) [66]. Nelde et al. identified nucleocapsid-specific antibodies in children using the well-validated Meso scale discovery (MSD) technique. However, it was remarked that immune responses against spike protein predominated. This observation is surprising since the N protein is abundant within the SARS-CoV-2 virion. Thus, the amplitude of the N-specific reaction might reflect a peak in viral load. However, the virus levels in the upper airways of children and adults are comparable at the point of initial infection [66].

Increased innate immune responses in MIS-C children may also play a crucial role in restricting systemic replication, explaining why children have greater asymptomatic and mild disease rates than adults [67,68]. Although antibody levels are frequently linked with illness severity, none of the children or adults in this research presented with severe illness or required hospitalization.

Anderson et al. demonstrated that neutralizing SARS-CoV-2 antibodies highly correlated with anti-full length S, S-RBD, and N antigens IgG antibodies [69]. Additionally, children with mild disease possessed variable levels of neutralizing antibodies. In contrast, MIS-C patients had higher neutralizing antibody titers than severe COVID-19 children. The latter was consistent with the observed elevated serum IgG titers against whole S antigen and S-RBD in children with MIS-C. The authors concluded that this finding is attributable to a more extended period after viral infection in children with MIS-C than those children with severe COVID-19 [69].

**Figure 3:**
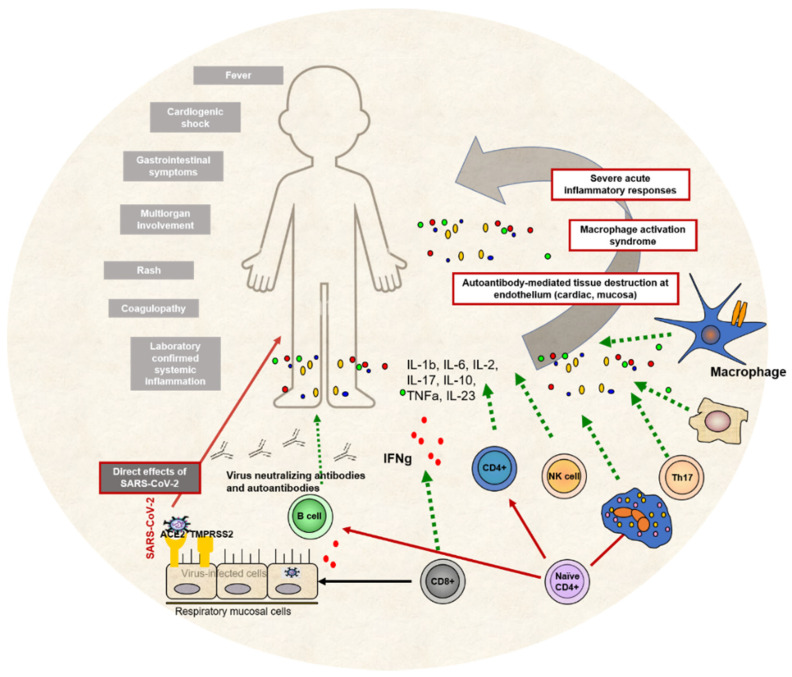
MIS-C immunological pathogenesis. Both innate and adaptive immune cells participate in various immune pathways leading to systemic inflammation, immune overactivation and antibody-mediated tissue destruction. The main clinical signs and symptoms of MIS-C are presented on the left side of the picture.

Immunological pathogenesis of MIS-C, including innate and adaptive immune responses and clinical presentation of the condition, can be seen in Figure 3.

## 3. Immune Response Profile and Long COVID-19 in Children

As the number of children affected by COVID-19 increased throughout the pandemic, evidence accumulated that long COVID can also be observed in childhood. The term long COVID covers the conditions where signs and symptoms of COVID-19 persist or are developed after acute disease and cannot be explained by other diagnoses.

It is well-accepted that overweight is associated with low-grade inflammation and disrupted immunological processes. Overweight is associated with impaired innate and adaptive cell types: helper T cells, cytotoxic T cells, B cells, and natural killer cells [70]. In addition, decreased production of antibodies and interferon γ is also reported among overweight patients [70]. This was observed in COVID-19 patients of all ages and could be connected with MIS-C.

To date, there is no clear agreement on the designation or duration for MIS-C. In contrast with the plenty of cases with long COVID in adults, the data are less conflicting in children. Many parents have observed fatigue, muscle pain or weakness, insomnia, general gastrointestinal issues, sore throat, cough, chest pain, fever, headache, concentration difficulties, loss of smell and taste in their children after COVID-19 [71,72]. Persistent symptoms are reported in up to 66%. Still, the major weaknesses of the existing studies are the small sample size and the lack of a control group. In a large Danish cohort study, the authors find that despite the high percentage of reported long COVID symptoms (12–51% depending on age), they are only 0.8% more frequent than the control group [73].

However, in children after COVID-19, loss of smell and taste, respiratory complaints, muscle weakness, fatigue, and chest pain are predominant. In contrast, the children in the control group reported significantly more often headaches, concentration difficulties, muscle and joint pain, fever, cough, nausea, and diarrhea [73]. In both groups, the symptoms increase with age. The burden of symptoms, measured as the number of simultaneous complaints, was higher among children with SARS-CoV-2 infection. In up to 75% of children, the symptoms resolved within 1–5 months [73].

Behnood et al. make a comprehensive meta-analysis of controlled and uncontrolled studies of persistent symptoms in children and young people after SARS-CoV-2 infection [74]. The frequency of most common persistent symptoms was comparable in SARS-CoV-2 positive cases and controls. The authors found that the risk for loss of smell, headache, cognitive difficulties, sore throat and eyes was significantly higher in post-COVID cases than in controls [74]. They also found that age was related to a higher incidence of all symptoms except cough.

The uniformity of these results suggests that the COVID-19 pandemic harms children not only through its infectious agent (SARS-CoV-2) but also psychologically.

## 4. Future Directions of Immunological Studies of MIS-C

MIS-C is a multiorgan hyperinflammatory disease that mimics some of the KD features. Based on this, most therapies in MIS-C are influenced by the well-established protocols in KD. However, despite some overlapping symptoms, both syndromes have marked differences in critical clinical, inflammatory, and autoantibody signatures [75]. Therefore, several antirheumatic drugs like monoclonal anti-IL-6 antibodies, IL-1 receptor antagonists, monoclonal anti-IL-1 antibody and monoclonal anti-TNF-α antibodies are a subject to current attention and research in studies, clinical trials and case reports. Still, the data are spare [76].

We urgently need to shed light on the different immune reaction scenarios in children with asymptomatic SAR-CoV-2 infection, COVID-19, severe COVID-19 and MIS-C. A clear and detailed picture of the MIS-C pathogenesis, especially intelligible clinically translated, could indicate the proper window to add agents that target the immune system. Molecular, genetic and immunological studies with a sufficient number of patients and various markers (including those associated with CDR3, HLA) can clarify pathogenetic mechanisms, describe shared features, and point specific diagnostic and prognostic approaches.

Of paramount importance to clinicians and parents is how to predict which child is at increased risk of developing MIS-C. As a rule, children with MIS-C are healthy and without concomitant diseases. However, the epidemiological studies demonstrate PCR detection of SARS-CoV-2 in nasopharyngeal smear in approximately 30% of MIS-C cases [77]. This finding is strong evidence of possible postinfectious delayed hyperinflammatory immune response against the virus [77]. Additionally, Porritt et al. describe a significant TRBV11-2 expansion supported by S-antigen-like T cell skewing, allowing them to recommend future studies of the SAg-like motif that could accelerate new therapeutic and prevention opportunities for patients with severe COVID-19 and MIS-C [77]. Expanding and amplifying knowledge could help match the puzzle and find the clue for etiological/pathogenetic and individualized treatment or prevention.

## 5. Conclusions

Many studies demonstrated that children possess higher protection against SARS-CoV-2 (especially concerning alfa and delta strains) than adults. A possible explanation is the generally vigorous and prolonged anti-SARS-CoV-2 adaptive immune response and immune cross-reactivity against other human coronaviruses. This apparent childhood advantage could be the clue to elucidate the immunological pathways involved in severe COVID-19, MIS-C, long COVID, etc.

In summary, there are three main hypotheses about the possible immunological pathogenetic pathways that initiate and drive MIS-C development: genetic predisposition concerning the ability of T cells to bind to S-protein; direct viral effect leading to endothelial dysfunction, platelet activation and multiorgan damage and the leading theory of maladaptive postinfectious response at different levels—APCs-T cells interactions, antigen-antibody-mediated cytokine storm, superantigen Vβ31-3T cells activation, formation of immune complexes or autoantibodies, etc.

The need for creating a realistic picture of the immunological puzzle of SARS-CoV-2 infection in childhood does not arise only from pure scientific interest. However, it is dictated by the urgency for defining adequate diagnostic criteria, treatment algorithms and prevention strategies—vaccine prophylaxis and predicting high-risk, genetic or immunologic predisposed patients.

## Figures and Tables

**Table 1:** Criteria for MIS-C diagnosis according to World Health Organization (WHO) [5], Centers for Disease Control and Prevention (CDC) [6] and Royal college of pediatrics and child health (RCPCH, UK) [7] recommendations.

World Health Organization	Centers for Disease Control and Prevention (US)	Royal College of Pediatrics and Child Health (UK)
Six of 6 criteria must be met: 1. Age 0 to 19 years 2. Fever more than 3 days 3. Clinical signs of multisystem involvement (at least 2 of the following): hypotension or shock cardiac dysfunction, pericarditis, valvulitis or coronary abnormalities (including echocardiographic findings or elevated troponin/BNP) Rash, bilateral nonpurulent conjunctivitis, or mucocutaneous inflammation signs (oral, hands, or feet) Acute gastrointestinal symptoms (diarrhea, vomiting, or abdominal pain) Evidence of coagulopathy (prolonged PT or PTT; elevated D-dimer 4. Elevated markers of inflammation (e.g., ESR, CRP, or procalcitonin) 5. Excluding microbial cause of inflammation (bacterial sepsis and staphylococcal/streptococcal) toxic shock syndromes 6. Any of the following evidence of SARS-CoV-2 infection: RT-PCR, serology, antigen test, contact with a COVID-19 positive subject	1. Age < 21 years 2. Presenting with Fever *, laboratory evidence of inflammation **, and evidence of clinically severe illness requiring hospitalization, with multisystem (>2) organ involvement (cardiac, renal, respiratory, hematologic, gastrointestinal, dermatologic or neurological); 3. No alternative plausible diagnoses; AND 4. Evidence for SARS-CoV-2 infection (recent or current) laboratory (RT-PCR, antigen test; serology) or epidemiology (exposure to COVID-19 four weeks before the symptoms onset	1. Persistent fever > 38.5 °C 2. Evidence of single or multiorgan dysfunction: oxygen requirement, hypotension, abdominal pain, confusion, conjunctivitis, cough, diarrhea, headache, lymphadenopathy, mucus membrane changes, neck swelling, rash, respiratory symptoms, sore throat, swollen hands and feet, syncope, vomiting 3. Laboratory: abnormal fibrinogen, absence of potential causative organisms (other than SARS-CoV-2), hypoalbuminemia, lymphopenia, neutrophilia in most-normal neutrophils in some children; 4. Coagulopathy: high D-dimers, high ferritin, high IL-10 (if available), elevated IL-6 (if available) ***, high CRP neutrophilia, proteinuria, raised CK, raised LDH, raised triglycerides, raised troponin, thrombocytopenia, transaminitis 5./ECG and cardiac ultrasound/–evidence for cardiac involvement-myocardial, valves, pericardial, coronary arteries Chest X-ray—typical infiltrates, pleural effusion Abdominal ultrasound—ascites, lymphadenopathy, colitis, ileitis, hepatosplenomegaly Chest CT scan—native-typical lungs and pleura signs; with contrast-possible coronary artery abnormalities

* Fever > 38.0 °C for more than 24 h, or subjective fever lasting more than 24 h. ** Including, but not limited to, one or more of the following: an elevated C-reactive protein (CRP), erythrocyte sedimentation rate (ESR), fibrinogen, procalcitonin, d-dimer, ferritin, lactic acid dehydrogenase (LDH), or interleukin 6 (IL-6), elevated neutrophils, reduced lymphocytes and low albumin; *** Where IL-6 is not available, use CRP as a surrogate. BNP—brain natriuretic peptide; PT—prothrombin; PTT—partial thromboplastin time; ESR—erythrocyte sedimentation ratio; CRP—C-reactive protein; RT-PCR—reverse transcription polymerase chain reaction; CK—creatine kinase; LDH—lactate dehydrogenase; ECG—electrocardiography; CT—computerized tomography.

**Table 2:** Receptors associated with SARS-CoV-2 infection in children and adults [23,24,25,26,27].

**Expression** **Endosomal Surface**	**Name**	**Function**	**Associated Transcription Factors**	**Features**
	TLR3	Recognition of dsRNA, produced during viral RNA replication	NF-κB IRF7 IRF3	- TLR7 gene mutation with loss-of-function decreases the production of IFN; - imiquimod can boost the respiratory pathogen′s innate immune defense
	TLR7	Detection of ssRNA		
	TLR8	Detection of ssRNA		
	TLR9	incites by DNA viruses that contain unmethylated CpG DNA		
Cell surface	TLR1 TLR2 TLR4 TLR5 TLR6	Recognition of viral nucleic acids		- Increased expression of TLR4 in COVID-19 patients; - In an animal ARDS model, the SARS-CoV2 provoked TLR4 mutation is linked with decreased lung injury; In a mice model of infected respiratory lungs (SARS and H1N1), the TLR4 mobilizations depend on oxidized phospholipids′ pulmonary expression
Cytosol	RIG I	Detection of RNA containing 5′-triphosphate residue		- RLR downstream molecule depletion, MAVS, diminish inflammatory cytokines synthesizing (IL-6, TNF-α, IFN-γ, MIP-1α, RANTES, IP-10) - in vitro studies of SARS-CoV and MERS-CoV infections indicate RIG-I and MDA-5 upregulation
	MDA-5	long dsRNA		- SARS-CoV or MERS-CoV infection leads to RIG-I and MDA-5 upregulation - MDA5 and LGP2 have main cytosol and weak epithelial surface expression; when exposed to INF type I or III demonstrate intense upregulation through positive feedback regulation.
	DC-SIGN; L-SIGN	C-type lectins; bind to carbohydrate residues; recognize the S protein RBD		- DC-SIGN binds the viruses and mediates their interaction with the ACE-2 expressing target cells; - L-SIGN and DC-SIGN could serve as an alternative to the primary ACE-2 SARS-CoV-2 receptor; - DC-SIGN mediates the physical interaction between DCs cells and T lymphocytes
	ACE-2	Binding to RBD domain of S protein		- Age-dependent expression; - widely distributed on macrophages - high expression on alveolar macrophages;

TLR—toll-like receptor; RIG-I—retinoic acid-inducible gene 1; MDA-5—melanoma differentiation-associated protein 5; DC-SIGN—dendritic cell-specific intercellular adhesion molecule-3-grabbing non-integrin; L-SIGN—liver/lymph node-specific intercellular adhesion molecule-3-grabbing integrin; ACE-2—angiotensin-converting enzyme; NF-κB—nuclear factor κB; IRF3, IRF7—interferon response factor 3 or 7; RBD—receptor-binding domain; ARDS—acute respiratory distress syndrome; RLRs—retinoic acid-inducible gene 1 (RIG-I)–like receptors;.

**Table 3:** Role of monocytes and macrophages during SARS-CoV-2 infection in children and adults [23,35,36,37,38,39,40].

Type	Function
M1 macrophages	M1 macrophages present antigens to T cells, inhibit tumor growth, produce proinflammatory cytokines such as IL-6, IL-12, and TNF-α [35] M1 decreases during SARS-CoV-2 infection through apoptosis and necrosis [38]. On the other hand, the overactivation of M1 macrophages contribute to the development of severe disease course [23]
M2 macrophages	M2 macrophages are the key player in tissue repair and wound healing by producing anti-inflammatory cytokines such as IL-10 and TGF-β [36,37]
Classical monocytes CD14++CD16−	Phagocytic cells, which absorb pathogens; production of proinflammatory cytokines; activation of other immune cells [38]
Non-classical monocytes CD14+CD16++ SLAN (glycosylated expression form of P-selective glycoprotein-1 (PSGL-1)	SLAN + non-classical monocytes (type 2) are undetectable in both moderate and severe patients with COVID-19, while non-classical type 1 monocytes are elevated [23]
Intermediate monocytes CD14++CD16+	Release of inflammatory cytokines—IL-1β, IL-6, IL-8 and TNF-α [38,39] Severe disease manifestation [23]
CD56+CD14+Ki67+IFN-γ+ monocyte	Produce IFN-g and Granzyme B in patients with moderate and severe COVID-19 [40]
